# Utility of Salivary Cortisol and Cortisone in the Diagnostics of Adrenal Insufficiency

**DOI:** 10.1210/clinem/dgae486

**Published:** 2024-07-12

**Authors:** Kåre Kvam Hellan, Martin Lyngstad, Paal Methlie, Kristian Løvås, Eystein Sverre Husebye, Grethe Åstrøm Ueland

**Affiliations:** Department of Clinical Science, University of Bergen, 5009 Bergen, Norway; Department of Clinical Science, University of Bergen, 5009 Bergen, Norway; Department of Clinical Science, University of Bergen, 5009 Bergen, Norway; Department of Medicine, Haukeland University Hospital, 5021 Bergen, Norway; Department of Medicine, Haukeland University Hospital, 5021 Bergen, Norway; Department of Clinical Science, University of Bergen, 5009 Bergen, Norway; Department of Medicine, Haukeland University Hospital, 5021 Bergen, Norway; Department of Medicine, Haukeland University Hospital, 5021 Bergen, Norway

**Keywords:** adrenal insufficiency, salivary cortisol, diagnostic performance, cosyntropin test

## Abstract

**Background:**

Salivary cortisol (sa-cortisol) and salivary cortisone correlate well with serum cortisol (s-cortisol) but validated reference ranges for healthy individuals are lacking.

**Objective:**

To establish cutoff levels for sa-cortisol and cortisone following cosyntropin testing and assess their diagnostic utility in adrenal insufficiency (AI).

**Methods:**

Steroids in saliva were assayed using liquid chromatography tandem mass spectrometry before and after administration of a 250-µg cosyntropin test in 128 healthy subjects (16 on oral estrogens) and 59 patients with suspected AI, of whom 26 were diagnosed with AI with conventional serum cortisol criteria. The cutoff level for AI was defined as the 2.5th percentile in healthy subjects not receiving estrogens. Performance was evaluated by calculating diagnostic accuracy and analyzing receiver operating characteristic curves.

**Results:**

The sa**-**cortisol cutoff 60 minutes after cosyntropin stimulation was 12.6 nmol/L (accuracy 89%, sensitivity 85%, and specificity 90%). Salivary cortisone and the sum of sa-cortisol and cortisone exhibited poorer diagnostic performance than sa-cortisol. The correlation between sa-cortisol and s-cortisol was best described by a model incorporating 2 regression lines (*R*^2^ = 0.80). Segmented regression analysis identified a breakpoint at sa**-**cortisol 9.7 nmol/L and s-cortisol 482 nmol/L, likely corresponding to saturation of cortisol binding globulin. Healthy subjects on oral estrogens demonstrated a linear agreement between s- and sa-cortisol through all measurements. Seventeen healthy subjects repeated the test, with similar outcomes, but reproducibility in terms of intraclass coefficient and correlation was poor.

**Conclusion:**

Sa-cortisol in cosyntropin-test has high diagnostic accuracy in detecting adrenal insufficiency and is particularly useful in women on oral estrogens. An sa**-**cortisol ≥ 12.6 nmol/L assayed with liquid chromatography tandem mass spectrometry 60 minutes after 250 µg cosyntropin is normal.

The standard cosyntropin stimulation test is the most commonly used dynamic test for adrenal function, be it primary, secondary, or tertiary. The principle of the cosyntropin test is stimulation of the adrenal cortex by injection of a synthetic analogue of ACTH (ACTH_1-24_) called cosyntropin (1). The test should be performed in the supine position, and the cortisol response is measured 30 and 60 minutes after IV injection. When analyzed using liquid chromatography tandem mass spectrometry (LC-MS/MS), a normal response is considered to be serum cortisol (s-cortisol) above 412 nmol/L after 30 minutes, and 485 nmol/L after 60 minutes (2). Patients on oral estrogens are at risk of obtaining a false-positive test results because of increased cortisol binding globulin (CBG) level (3). Basal morning s-cortisol levels, and home waking saliva (sa)-cortisone sampling are good alternatives to the cosyntropin test for diagnosing adrenal insufficiency (4-9).

Salivary cortisol (sa-cortisol) measurements are commonly used in the diagnostics of Cushing syndrome, but their role in the diagnostics of adrenal insufficiency (AI) remains to be established. The correlation between s-cortisol and sa-cortisol is very high (10), and this extends to dynamic diagnostic tests such as dexamethasone suppression test (11) and the cosyntropin stimulation test (12). Indeed, the correlation between s-cortisol and sa-cortisone is even more pronounced, likely because the extensive expression of 11 beta-hydroxysteroid dehydrogenase type 2 (11βHSD type 2) in the salivary glands.

This enzyme catalyzes a rapid conversion of cortisol to cortisone upon its transfer to saliva.

Sa-cortisol and sa-cortisone represents the free and biologically active cortisol and their levels are not influenced by oral estrogens. Their use to evaluate the response to cosyntropin has been evaluated in small studies and with various analytical assays (12-16). Cutoffs have been defined but were not validated for LC-MS/MS in adequately sized cohorts. Here, we tested the diagnostic accuracy of the cosyntropin test using sa-cortisol and sa-cortisone assayed by LC-MS/MS in an adequately sized sample of healthy subjects and tested its performance in persons with suspected AI.

## Methods

### Study Population

From June 2016 to August 2017 we consecutively included patients referred for evaluation of AI for a cosyntropin testing (n = 59). We also recruited healthy subjects among medical students and hospital and university staff (n = 128), of which 16 used oral estrogens (Table 1). For evaluation of reproducibility, the test was repeated in 17 healthy subjects. All participants performed a nonfasting cosyntropin test between 8 and 12 Am. Serum and saliva were collected at baseline, and after 30 and 60 minutes. Data derived from the serum samples have been published previously (2). Patients using cortisone acetate did not take their morning medication on the day of the test, and prednisolone users were without medication for at least 72 hours. Patients tested for AI because of long-term use of opioids were opioid abstinent on the day of testing. Abnormal test results prompted further testing according to European guidelines for diagnosing AI (17). Patients were finally diagnosed as having primary adrenal insufficiency (PAI) (n = 6), secondary adrenal insufficiency (SAI) (n = 20), or being healthy (n = 33), consistent with classification in the original study.

**Table 1.** Characteristics of patients and healthy subjects

	Healthy subjects*^a^*	Suspected adrenal insufficiency
Patients, n	112	59
Women, n (%)	62 (55)	35 (59)
Age, y (range)	40 (23-68)	49 (19-86)
Systolic blood pressure, mm Hg, (range)	131 (109-170)	121 (85-181)
Diastolic blood pressure, mm Hg, (range)	76 (58-99)	76 (60-105)
Smokers, n (%)	3 (3)	14 (24)
Body mass index, kg/m^2^ (range)	24 (16-34)	25 (18-49)
Creatinine µmol/L (range)	72 (50-100)	68 (47-244)
Estimated glomerular filtration rate mL/min/1.73/m^2^ (range)	102 (69-120)	101 (7-120)

Categorical data are given as n (%); continuous data are given as median (range).

^*a*^Subjects on oral estrogens excluded.

### Glucocorticoid Assay

Serum samples were assayed for cortisol by LC-MS/MS as described in detail previously (18). Total analytical variation (coefficient of variation) for cortisol was <7.4 (97% accuracy). The LC-MS/MS laboratory participates in an external quality control program (NEQAS for serum steroids). Additionally, the accuracy of our LC-MS/MS assay is regularly verified by analyzing certified reference samples, with the accuracy according to the materials is 105% for cortisol. Saliva samples were analyzed by LC-MS/MS at the Core Facility for Metabolomics, University of Bergen, Norway. The method is previously described in detail (19).

### Statistics

The subject characteristics are reported as median (range). Because of the nonnormal distribution of the data, cutoffs for sa-cortisol and sa-cortisone after cosyntropin were determined using nonparametric statistical methods. The 2.5th percentile of sa-cortisol and sa-cortisone at baseline, as well as after 30 and 60 minutes, in healthy control subjects not using oral estrogens, were established as the lower normal limit for the test. The 2.5th percentile is reported with a 90% CI, adhering to recommendations for cutoffs derived from upper or lower percentiles (20). The diagnostic accuracy, the sensitivity and specificity, were calculated for sa-cortisol and sa-cortisone 30 and 60 minutes after cosyntropin stimulation. Positive predictive value (PPV) and negative predictive value (NPV) were calculated in the patient cohort. The Mann-Whitney *U* test was used to compare groups. Receiver operating curves (ROC) were also employed to validate the defined cutoff levels, and if necessary, identify more optimal cutoff levels. Spearman's *ρ* was calculated for sa-cortisol and sa-cortisone measured with repeated testing in 17 healthy subjects, and to assess the correlation between sa-cortisol, sa-cortisone, and various clinical and biochemical parameters. The reproducibility for sa-cortisol and sa-cortisone was also assessed by determination of intraclass correlation coefficient (ICC) (95% CI) using In-transformed data and a random-effects mixed model with participant identification as the random variable. An ICC <0.40 was considered as poor reproducibility, 0.40 to 0.75 as fair-to-good reproducibility, and ≥0.75 as excellent reproducibility (21). The significance level was set at *P* < .05.

### Ethics

The study was approved by the local ethics committee (Rek nr:28179), and all participants signed the informed consent form.

## Results

### Sa-cortisol and Cortisone in Heathy Subjects

The 112 healthy subjects not using oral estrogens were used to test the utility of salivary cortisol and cortisone in cosyntropin testing. The medians and ranges at baseline and 30 and 60 minutes are given in Table 2. The 2.5th percentiles for sa-cortisol and sa-cortisone are shown in Table 2.

**Table 2.** Median (range) and 2.5th percentile (90% CI) in nmol/L for sa-cortisol and sa-cortisone in healthy control subjects

	Saliva-cortisol			Saliva-cortisone		
	Baseline	30 minutes	60 minutes	Baseline	30 minutes	60 minutes
Healthy controls*^a^* nmol/L, median (range)	4.0 (0.4-34.9)	23.0 (4.8-65.3)	36.0 (10.8-89.3)	20.5 (5.4-80.3)	50.0 (14.4-91.5)	68.5 (21.1-121.5)
Men	4.4 (0.7-22.5)	22.2 (5.1-65.3)	35.9 (10.8-89.3)	22.1 (5.6-45.1)	51.0 (18.0-86.0)	68.9 (21.1-121.5)
Women	3.7 (0.4-34.9)	23.1 (4.8-47.7)	36.7 (12.4-71.6)	19.0 (5.4-8.3)	49.6 (14.4-91.5)	67.2 (30.1-114.0)
Age range 20-40	4.6 (0.4-34.9)	23.1 (10.5-50.0)	36.5 (10.8-71.6)	23.6 (5.4-80.3)	52.0 (26.2-91.5)	68.5 (21.1-114.0)
41-60	3.7 (0.7-22.5)	22.5 (4.8-65.3)	35.4 (12.4-89.3)	18.5 (5.6-38.1)	49.2 (14.4-82.1)	67.8 (34.6-121.5)
61-80	3.5 (1.1-5.4)	22.4 (5.1-36.0)	36.0 (23.8-53.5)	18.0 (8.9-31.9)	50.1 (18.0-86.0)	81.5 (47.6-119.8)
2.5th percentile nmol/L (90% CI)	0.7 (0.4-1.1)	8.1 (4.8-11.1)	12.6 (10.8-18.1)	5.8 (5.4-7.4)	24.4 (14.4-30.6)	32.4 (21.1-39.6)
Healthy controls on estrogen	5.8 (1.4-25.4)	19.1 (10.4-36.3)	35.9 (16.1-56.8)	24.0 (11.9-60.5)	45.4 (32.7-73.2)	69.8 (44.3- 96.4)

^*a*^Not on oral estrogen.

All healthy controls showed increased sa-cortisol levels from 0 to 60 minutes. Two healthy subjects did not exhibit a further increase from 30 to 60 minutes.

The median increase in sa-cortisol and sa-cortisone from 0 to 60 minutes was 31.3 nmol/L (range, 2.9-69 nmol/L) and 46.7 nmol/L (range, 43.3-49.4 nmol/L), respectively. The corresponding 2.5^th^ percentile for the increase in sa-cortisol and sa-cortisone was 8.1 nmol/L (90% CI, 2.9-12.9) and 11.9 nmol/L (90% CI, −2.8 to 22.9), respectively (Supplementary Table S1 (22)). We did not find significant differences in responses between men and women, or between age groups (Supplementary Table S2 (22)). Furthermore, there were no significant correlations between sa-cortisol or sa-cortisone and age, body mass index or kidney function (measured both as creatinine and estimated glomerular filtration rate at any timepoints (Supplementary Table S3 (22)).

### Reproducibility of the Test

The cosyntropin test was repeated in 17 healthy subjects. The mean time interval between the repeated tests was 379 (range, 28-391) days. Wilcoxon signed-rank test for related samples revealed no significant differences between sa-cortisol in the 2 tests at baseline (*P* = .084), 30 minutes (*P* = .64), or after 60 minutes (*P* = .95). Sa-cortisol levels in the 2 tests revealed no significant correlation at baseline (*ρ* = 0.074; *P* = .77), 30 minutes (*ρ* = 0.054; *P* = .04), or 60 minutes (*ρ* = 0.31; *P* = .10). For sa-cortisone the baseline values were statistically significantly different (*P* = .013), but not after 30 and 60 minutes (*P* = .51 and *P* = .78, respectively). For sa-cortisone there was also a significant correlation between tests 60 minutes post cosyntropin (*ρ* = 0.58; *P* = .014). Correspondingly, the within-subject reproducibility in terms of ICC was only significant for sa-cortisone at 60 minutes (ICC 0.81; 95% CI, 0.47 −0.93).

### Correlation of s-cortisol With sa-cortisol and sa-cortisone

The correlations between s-cortisol and sa-cortisol were 0.81, 0.43, and 0.44 (*P* < .05), respectively, and between s-cortisol and sa-cortisone 0.72, 0.36, and 0.46 (*P* < .05), respectively. To account for saturation of CBG, we employed segmented regression for serum and saliva cortisol utilizing all timepoints from all healthy subjects, excluding those on oral estrogens.

**Figure 1.**
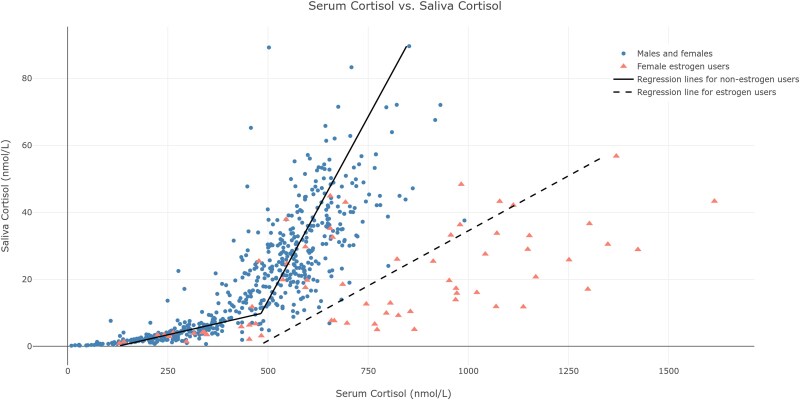
Segmented regression for the correlation between saliva and serum cortisol at all timepoints (basal, 30, and 60 minutes after stimulation with cosyntropin). A model with 2 regression lines shows the best fit to the model with *R*^2^ = 0.80.

This analysis identified a breakpoint in the curve at sa-cortisol 9.7 nmol/L and s-cortisol 482 nmol/L (Fig. 1). A model with 2 regression lines produced a fit with a coefficient of determination *R*^2^ of 0.80. The 16 individuals using oral estrogens did not reveal the breakpoint in the curve, but rather had a linear correlation between serum and saliva cortisol (Fig. 1).

### Healthy Subjects on Oral Estrogens

The 16 healthy subjects on estrogen had median sa-cortisol of 5.8 nmol/L (range, 1.4-25.4 nmol/L), 19.1 nmol/L (range, 0.4-36.3 nmol/L), and 35.9 nmol/L (range, 16.1-56.8 nmol/L) at baseline and 30 and 60 minutes, respectively (Table 2). The median levels were not significantly different from healthy controls not using oral estrogens. The median sa-cortisone levels of the participants on oral estrogens at baseline, 30 and 60 minutes respectively, are reported in Table 2. The median increase from 0 to 60 minutes was 27.6 nmol/L (range, 12.9-46.9 nmol/L). For sa-cortisone the median level in estrogen users was 38.9 nmol/L (range, 18.7-80.1 nmol/L).

### Diagnostic Performance for sa-cortisol and sa-cortisone in AI

**Figure 2.**
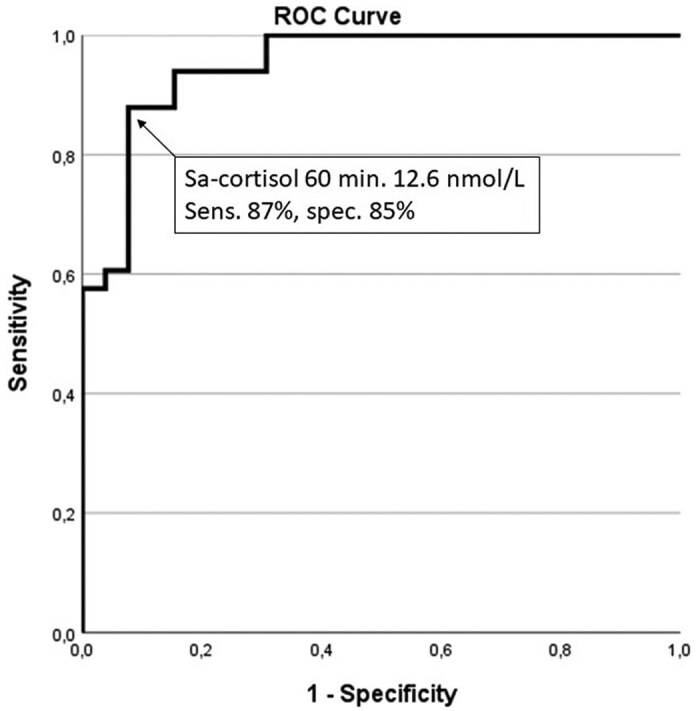
ROC curve for sa-cortisol 60 minutes after stimulation with cosyntropin. The area under the curve is 0.95.

A total of 59 patients were tested for suspected AI, of whom 26 finally had the diagnosis confirmed based on serum cortisol response (6 had PAI and 20 SAI). The causes of suspected AI and finally diagnosed AI are shown in Table 3. Applying the 2.5th percentile after 30 minutes as cutoff for sa-cortisol (8.1 nmol/L), yielded a sensitivity of 88%, specificity of 85%, PPV of 82%, NPV of 90%, and diagnostic accuracy for AI of 86% (Supplementary Table S4 (22)). Applying the 2.5th percentile after 60 minutes as cutoff for sa-cortisol (12.6 nmol/L), resulted in a sensitivity of 85%, specificity of 90%, PPV of 88%, and NPV of 88%, with a diagnostic accuracy of 89% for diagnosing AI. ROC curve for sa-cortisol after 30 minutes displayed an area under the curve (AUC) of 0.93 (95% CI, 0.87-0.97). The defined cutoff for sa-cortisol at 30 minutes (8.1 nmol/L) achieved an optimal sensitivity of 85% and a specificity of 88% for the diagnosis of AI. For sa-cortisol after 60 minutes, the corresponding AUC was 0.95 (95% CI, 0.89-1.0), sensitivity 87%, and specificity 85%, based on optimum cutoff for 12.6 nmol/L (Fig. 2).

**Table 3.** Overview of patients with suspected AI in the study, and their final diagnosis

Indication for cosyntropin testing	Suspected AI, n = 59	Diagnosed AI, n = 26
PAI	13	6
SAI	46	20
- Pituitary adenoma	9	4
- Insufficiency due to the use of glucocorticosteroids	14	9
- Chronic opiate treatment	2	1
- Unilateral adrenalectomy for autonomous cortisol secretion	6	5
- Unknown etiology	15	1

Abbreviations: AI, adrenal insufficiency; PAI, primary adrenal insufficiency; SAI, secondary adrenal insufficiency.

Applying the 2.5th percentile after 30 minutes as cutoff for sa-cortisone (24.4 nmol/L), resulted in a sensitivity of 65%, specificity of 97%, PPV of 94%, and NPV of 78%, with a diagnostic accuracy for AI of 83% (Supplementary Table S4 (22)). Applying sa-cortisone after 60 minutes (32.4 nmol/L), gave a sensitivity of 69%, specificity 97%, PPV 95%, and NPV of 78%, and a diagnostic accuracy for AI of 85% (Supplementary Table S4 (22)). The ROC curve for sa-cortisone 30 minutes after stimulation exhibited an AUC of 0.96 (95% CI, 0.91-1.0). This was observed despite the sa-cortisone (24.4 nmol/L) cutoff yielding a low diagnostic accuracy for AI. Analysis of the ROC curve indicated that an optimal cutoff for sa-cortisone measured after 30 minutes would be 32.8 nmol/L, achieving a sensitivity of 91% and specificity of 92%. After 60 minutes the AUC was 0.96 (95% CI, 0.91-1.0), and the optimal cutoff 45.4 nmol/L, giving a sensitivity of 91% and a specificity of 92%.

Six of the patients with suspected AI used oral estrogens, of whom 2 were finally diagnosed with AI. They all exhibited a response in sa-cortisol and cortisone at 30 and 60 minutes after cosyntropin stimulation consistent with their final diagnosis. Moreover, the 2 diagnosed with AI had an increase in sa-cortisol and cortisone below the increase cutoff in healthy subjects without estrogens. One of the referrals found healthy had an increase of sa-cortisol below cutoff (7.2 nmol/L) after 30 minutes but had a normal increase after 60 minutes. They all achieved adequate increase in sa-cortisone. Using the sum of sa-cortisol and sa-cortisone did not improve the diagnostic performance for AI after cosyntropin test (data not shown).

## Discussion

This study establishes LC-MS/MS-based cutoff levels for sa-cortisol and sa-cortisone after cosyntropin test for the diagnostics of AI. Sa-cortisol 60 minutes after cosyntropin stimulation gave the best diagnostic accuracy. We also calculated a cutoff for the lowest expected increase in sa-cortisol and cortisone, which can be applied as additional criteria for positive or negative tests, when in doubt. Given that our analytical assay is traceable to international reference materials (23), our cutoffs may, with minor adjustments, be applicable for all laboratories measuring sa-cortisol and sa-cortisone by LC-MS/MS, in line with 2 earlier reports (15, 16).

Two major factors may influence the correlation between s-cortisol and sa-cortisol and cortisone, namely, CBG level and 11βHSD type 2 activity. First, the saturation of CBG occurs between serum cortisol levels of 400 to 500 nmol/L depending on the patient's CBG levels (24). For individuals not using oral estrogens, we found that the correlation between sa-cortisol and s-cortisol was best described by a model incorporating two regression lines. This model identified a breakpoint for s-cortisol, which likely corresponds to the saturation of CBG, with steeper increase in sa-cortisol for serum levels above that level. Notably, this relationship was different in individuals using oral estrogens, who have higher CBG levels, exhibiting linearity across the entire range of measurements. For oral estrogen users, we found that the median levels of sa-cortisol and cortisone were similar to nonusers. For serum samples, we showed in our previous study that the median s-cortisol levels in estrogen users were nearly double those without oral estrogens (2). This is in line with findings from a recent Swedish study of saliva measurements after cosyntropin test in healthy controls with and without oral estrogens (25). All healthy subjects on oral estrogens had an adequate increase of sa-cortisol and sa-cortisone, and all the patients on oral estrogens tested for AI had an increase corresponding to their final diagnosis. We therefore conclude that sa-cortisol and/or cortisone can be used to evaluate the response to cosyntropin in oral estrogen users.

Second, the high activity of the enzyme 11βHSD type 2 can also affect the correlation between s-cortisol and sa-cortisol. Previous studies have shown that s-cortisol correlates better with sa-cortisone than sa-cortisol (13, 14), but our data do not support this finding. In the original study on serum samples, we found an excellent test reproducibility for the 20 healthy controls repeating the test (2). However, the saliva data did not exhibit the same degree of reproducibility. The explanation for the low reproducibility probably is that sa-cortisol is more responsive to intercurrent events and timing of the sample collection in relation to the intrinsic biorhythms compared to s-cortisol. For stimulated values the degree of 11βHSD type 2 conversion of sa-cortisol to cortisone could vary significantly and affect how much sa-cortisol that is actually converted to cortisone. For the 60-minute cortisone level on the other hand, the upper rate of cortisol to cortisone conversion by 11βHSD type 2 is potentially limiting, providing higher ICC. Despite these factors, all individuals that repeated the test were classified as healthy at both test occasions.

Saliva secretion decreases with age (26), which could potentially impact the test results. However, we found no correlations between sa-cortisol or cortisone and age, and no differences between men and women. These findings support that age or sex-specific cutoffs for saliva cortisol or cortisone are not required (13).

Even though early morning s-cortisol shows large inter- and intra-individual variability, some advocate that early morning s-cortisol could be an alternative to the cosyntropin test, or at least a good screening test (6, 7, 9). Also, wakening sa-cortisone has shown good diagnostic performance in for adrenal insufficiency (4, 5, 8). Recently, a nasal formulation of cosyntropin has been developed, which could, combined with salivary cortisol measurement, make the cosyntropin test ambulatory (27).

One important strength of this study is the design where cutoffs for sa-cortisol and sa-cortisone was defined in a large cohort of healthy individuals and subsequently verified with ROC analyses in an independent cohort of patients with suspected AI. Previous studies have been small and underpowered (12-14). Moreover, we included individuals using oral estrogens, both in the healthy subject and in the suspected AI group. Finally, our analytical approach using segmented regression takes into account the saturation of CBG.

The study has some limitations. Few healthy controls who had a baseline sa-cortisol measured between 8 and 10 Am. As such, the proposed cutoff for morning sa-cortisol needs to be confirmed in larger cohort of patients with suspected AI. The number of persons on estrogens are also, relatively low. Finally, patients using cortisone acetate, prednisolone, or opioids may have not abstained from their medications long enough before the cosyntropin test, but the time intervals chosen were the longest found ethically justifiable.

In conclusion, sa-cortisol assayed on LC-MS/MS demonstrates excellent diagnostic performance both 30 and 60 minutes after cosyntropin stimulation. We propose 8.1 nmol/L at 30 minutes or12.6 nmol/L at 60 minutes as diagnostic cutoffs for the diagnosis of AI. The use of saliva cortisol in this setting will be particularly advantageous in patients with suspected AI using oral estrogens and those who pose challenges for venous cannulation.

## Data Availability

All original data generated and analyzed during this study are included in this published article.

## Abbreviations

11βHSD: 11 beta-hydroxysteroid dehydrogenase
AUC: area under the curve
CBG: cortisol binding globulin
ICC: intraclass correlation coefficient
LC-MS/MS: liquid chromatography tandem mass spectrometry
PAI: primary adrenal insufficiency
NPV: negative predictive value
PPV: positive predictive value
ROC: receiver operating characteristic
S-cortisol: serum cortisol
Sa-cortisone: salivary cortisone
SAI: secondary adrenal insufficiency
